# Big team science reveals promises and limitations of machine learning efforts to model physiological markers of affective experience

**DOI:** 10.1098/rsos.241778

**Published:** 2025-06-25

**Authors:** Nicholas A. Coles, Bartosz Perz, Maciej Behnke, Johannes C. Eichstaedt, Soo Hyung Kim, Tu N. Vu, Chirag Raman, Julian Tejada, Van-Thong Huynh, Guangyi Zhang, Tanming Cui, Sharanyak Podder, Rushi Chavda, Shubham Pandey, Arpit Upadhyay, Jorge I. Padilla-Buritica, Carlos J. Barrera Causil, Linying Ji, Felix Dollack, Kiyoshi Kiyokawa, Huakun Liu, Monica Perusquia-Hernandez, Hideaki Uchiyama, Xin Wei, Houwei Cao, Ziqing Yang, Alessia Iancarelli, Kieran McVeigh, Yiyu Wang, Isabel M. Berwian, Jamie C. Chiu, Dan-Mircea Mirea, Erik C. Nook, Henna I. Vartiainen, Claire Whiting, Young Won Cho, Sy-Miin Chow, Zachary F. Fisher, Yanling Li, Xiaoyue Xiong, Yuqi Shen, Enzo Tagliazucchi, Leandro A. Bugnon, Raydonal Ospina, Nicolas M. Bruno, Tomas A. D'Amelio, Federico Zamberlan, Luis R. Mercado Diaz, Javier O. Pinzon-Arenas, Hugo F. Posada-Quintero, Maneesh Bilalpur, Saurabh Hinduja, Fernando Marmolejo-Ramos, Shaun Canavan, Liza Jivnani, Stanisław Saganowski

**Affiliations:** ^1^ University of Florida, Gainesville, FL, USA; ^2^ Wrocław University of Science and Technology, Wroclaw, Województwo Dolnośląskie, Poland; ^3^ Adam Mickiewicz University, Poznan, Poland; ^4^ Stanford University, Stanford, CA, USA; ^5^ Chonnam National University, Gwangju, Jeollanam-do, Republic of Korea; ^6^ Delft University of Technology, Delft, Zuid-Holland, The Netherlands; ^7^ Federal University of Sergipe, Sao Cristovao, Sergipe, Brazil; ^8^ FPT University, Hanoi, Vietnam; ^9^ Harvard Medical School, Boston, MA, USA; ^10^ Independent Researcher, State College, PA, USA; ^11^ Indian Institute of Science Education and Research Bhopal, Bhopal, Madhya Pradesh, India; ^12^ Indian Institute of Technology Bombay, Mumbai, Maharashtra, India; ^13^ Institución Universitaria ITM, Medellín, Colombia; ^14^ Montana State University, Bozeman, MT, USA; ^15^ Nara Institute of Science and Technology, Ikoma, Nara, Japan; ^16^ New York Institute of Technology, Old Westbury, NY, USA; ^17^ Northeastern University—Boston Campus, Boston, MA, USA; ^18^ Princeton University, Princeton, NJ, USA; ^19^ The Pennsylvania State University, University Park, PA, USA; ^20^ Universidad Adolfo Ibanez, Penalolen, Chile; ^21^ Research Institute for Signals, Systems and Computational Intelligence, sinc(i), FICH-UNL, CONICET, Santa Fe, Argentina; ^22^ Universidade Federal da Bahia, Salvador, Brazil; ^23^ University of Buenos Aires, Buenos Aires, Argentina; ^24^ Tilburg University, Tilburg, Netherlands; ^25^ University of Connecticut, Storrs, CT, USA; ^26^ University of Pittsburgh, Pittsburgh, PA, USA; ^27^ University of Akron, Akron, OH, USA; ^28^ Flinders University, Adelaide, South Australia, Australia; ^29^ University of South Florida, Tampa, FL, USA

**Keywords:** big team science, machine learning, emotion, physiology, generalizability, affective computing

## Abstract

Researchers are increasingly using machine learning to study physiological markers of emotion. We evaluated the promises and limitations of this approach via a big team science competition. Twelve teams competed to predict self-reported affective experiences using a multi-modal set of peripheral nervous system measures. Models were trained and tested in multiple ways: with data divided by participants, targeted emotion, inductions, and time. In 100% of tests, teams outperformed baseline models that made random predictions. In 46% of tests, teams also outperformed baseline models that relied on the simple average of ratings from training datasets. More notably, results uncovered a methodological challenge: multiplicative constraints on generalizability. Inferences about the accuracy and theoretical implications of machine learning efforts depended not only on their architecture, but also how they were trained, tested, and evaluated. For example, some teams performed better when tested on observations from the same (vs. different) subjects seen during training. Such results could be interpreted as evidence against claims of universality. However, such conclusions would be premature because other teams exhibited the opposite pattern. Taken together, results illustrate how big team science can be leveraged to understand the promises and limitations of machine learning methods in affective science and beyond.

## 1. Introduction

All throughout their lives, people experience phenomenological states they call ‘emotions’ [1]. For at least a century, researchers have been interested in the extent to which these emotional states have physiological markers. For an emotion artificial intelligence (AI) industry recently valued at $20+ billion [2], these physiological markers may be the key to creating technology that can unobtrusively track and respond to humans’ emotions. For emotion theorists, these physiological markers may provide fundamental insights into the nature of emotions themselves. For example, many theorists posit that emotional experience has a physiological basis, often suggesting that it is partially or fully built off afferent feedback from the peripheral nervous system [3–18]. If true, such accounts provide clues not only about *how* emotional experiences arise but also about *why* such a capacity evolved (e.g. to monitor and regulate physiological states) [4,19].

Over the past couple of decades, researchers have increasingly turned to machine learning methods to capture, study and debate the nature of emotion physiology [20–25]. In the present work, we evaluated the promises and limitations of this approach via a big team science competition. The competition focused on modelling physiological markers of the most elementary component of emotional experience: core affective feelings of valence (pleasantness versus unpleasantness) and arousal (often defined as strength of physiological activity or level of energy) [26]. By crowdsourcing this task, we sought to efficiently identify and probe particularly promising approaches to capturing links between physiology and affective experiences. We further sought to do so in a manner that establishes a high degree of *commensurability* (i.e. comparability). To do so, we had researchers commit to the same sets of training, testing and evaluation procedures. Simultaneously, we examined the impact of non-commensurability by systematically varying many of these methodological decisions—i.e. having researchers engage with multiple training, testing and evaluation frameworks.

Our examination of commensurability was motivated by reviews indicating a general *lack* of commensurability in many machine learning efforts focused on emotion physiology [20]. As an illustrative example, consider the challenges of comparing two simple but influential studies by Picard *et al*. [27] and Haag *et al*. [28] (table 1).

**Table 1.** Past efforts to use machine learning to predict emotion reports from physiology are difficult to compare. (For example, Picard *et al*. [27] and Haag *et al*. [28] used different datasets, outcomes, benchmarks and testing procedures. The present work standardizes and/or systematically varies those methodological details.)

citation	dataset	outcome	benchmark	testing procedure
Picard *et al*. [27]	*n* = 1 subject completes emotion self-elicitation task	emotion targeted by the self-elicitation task	percentage of times the model correctly predicted which emotion was targeted	leave-one-out cross-validation
Haag *et al*. [28]	*n* = 1 subject views emotional photos	external ratings of emotional photos	percentage of times predicted value fell within a range of the external ratings	hold out validation
present work with 12 teams	*n* = 30 subjects view emotional videos	self-reported affect	absolute prediction error (compared to two baseline models)	across-subject, -emotion, -induction and -time validation

On one hand, Picard *et al*. [27] (i) used peripheral nervous system data from a single participant who completed an emotion self-elicitation task (e.g. guided imagery) to (ii) predict which of eight emotions were targeted by the task. They (iii) defined accuracy as the percentage of times the model correctly predicted which emotion was targeted, and (iv) tested their model using leave-one-out cross-validation. In this validation approach, data are divided into multiple non-overlapping sets that are iteratively used to train and test models. Using this approach, Picard *et al*. [27] concluded that their model achieved 81% classification accuracy.

On the other hand, Haag *et al*. [28] (i) used peripheral nervous system data from a single participant who viewed emotional photos, to (ii) predict how other participants rated those photos. They (iii) defined accuracy as the percentage of times the predicted value fell within a range of ratings from other participants, and (iv) tested their model using hold-out validation. In this validation approach, data are divided into two sets, one for training and one for testing. Using this approach, Haag *et al*. [28] concluded 90% and 97% accuracy for predicted valence and arousal reports, respectively.

In this illustrative example, it is tempting to conclude that Haag *et al*. improved upon Picard *et al*.’s work—or, more broadly, that the field’s methods for capturing links between emotion and physiology are improving. However, such claims are difficult to substantiate owing to a lack of commensurability: the two teams used different datasets, outcomes, performance benchmarks and model validation procedures [20,29]. It is certainly possible that Haag *et al*. [28] developed a superior model. However, it is also possible that they studied a simpler emotional context (emotional photos versus self-elicitation), focused on a simpler outcome (core affect prediction versus discrete emotion classification), used a more liberal benchmark (falling within a range of observer ratings versus identifying the targeted emotion) and/or used more rigorous testing procedures (hold out versus leave-one-out cross-validation).

If incommensurability makes it difficult to compare the performance of two machine learning efforts, it is perhaps not surprising that it also confounds attempts to connect such work to theory. Researchers are increasingly using machine learning methodology to weigh in on century-old debates about emotion, e.g. whether underlying patterns are biologically innate, emotion specific, similar across contexts and stable throughout the course of an emotional event [30]. Yet, one possibility is that machine learning researchers reach different conclusions about connections to theory simply because they approach the questions in different ways [31]. The present work evaluates such a possibility by systematically varying training, testing and evaluation procedures in a machine learning challenge involving 12 groups of researchers.

## 2. Methods

For the competition, we used the Continuously Annotated Signals of Emotion dataset [32]. This dataset contains moment-to-moment measures of affective experience and multi-modal physiology from 30 volunteer subjects recruited from the Institute of Robotics and Mechatronics in Germany (15 males, age 28.6 ± 4.8 years and 15 females, age 25.7 ± 3.1 years). These subjects encountered two video inductions of fear, boredom, relaxation and amusement. In total, there were eight inductions, each 2−3 min in length. These videos were initially selected based on their use in prior research, but participants’ affect reports provided further evidence of their effectiveness as emotion inductions [32].

 Throughout the video inductions, participants used a joystick to navigate a continuous, 9-point, two-dimensional grid measuring valence (i.e. pleasantness versus unpleasantness) on the *x*-axis and arousal (i.e. low energy versus high energy) on the *y*-axis. Simultaneously, multi-modal peripheral nervous system measurements were collected: electrocardiography, blood volume pulse, electrodermal activity, respiration, skin temperature and electromyography activity over the zygomaticus major, corrugator supercilii and trapezius muscles. Notably, both the affect reports and peripheral nervous system signals were collected moment-to-moment (20 and 1000 samples s^−1^, respectively). This provided teams with a large set of observations with high temporal resolution. For each participant, the original dataset contained approximately (i) 25 401 valence and arousal reports and (ii) 1 270 083 recordings of peripheral nervous system activity. Using these observations, teams were challenged to use peripheral nervous system features to predict affect reports via their preferred machine learning methodology.

### 2.1. Teams

For the competition, we sought to recruit up to 15 teams based on the availability of funds. Teams were eligible to participate if they agreed to (i) make their code openly available, (ii) collaborate on a manuscript describing the challenge results, and (iii) not cheat (e.g. look for the original dataset). Eighteen teams completed the application to join the challenge, which asked them to report previous experience with machine learning challenges, the number of papers published in this domain, their planned approach to the challenge and the CV of members of their teams. The competition organizers selected teams based on averaged ratings of their (i) experience with machine learning challenges (N.A.C.), (ii) expertise in emotion research (M.B.) and/or (iii) planned approach to the challenge (S.S.). These evaluations were made independently and then discussed as a group. Of the 15 teams that were invited to compete, three dropped out owing to difficulties encountered during the challenge. In total, 12 teams completed the challenge, using a variety of modelling approaches (electronic supplementary material, table S1) [33–35].

Teams received $300 for completing the competition. Those who developed approaches judged to be the most promising received a $200 bonus. This payment structure was designed to balance incentives for effort ($300 for all teams) and performance ($200 for most promising approaches). To subjectively identify the most promising approaches, two competition organizers (S.S. and B.P.) worked together to examine (i) how well the teams performed by inspecting their root mean square error, and (ii) if top performing, their underlying code. This two-step process allowed us to consider performance, perceived methodological soundness and reproducibility when choosing which models to further evaluate. This allowed us to efficiently select promising models for follow-up investigations: a selection process that helps conserve resources consumed by large-scale machine learning efforts [36].

### 2.2. Model training and testing

There were no constraints on how teams were instructed to model the data. For example, some teams deployed relatively simply tree-based ensemble models, whereas others deployed relatively complex deep learning techniques (electronic supplementary material, table S1).

**Figure 1.**
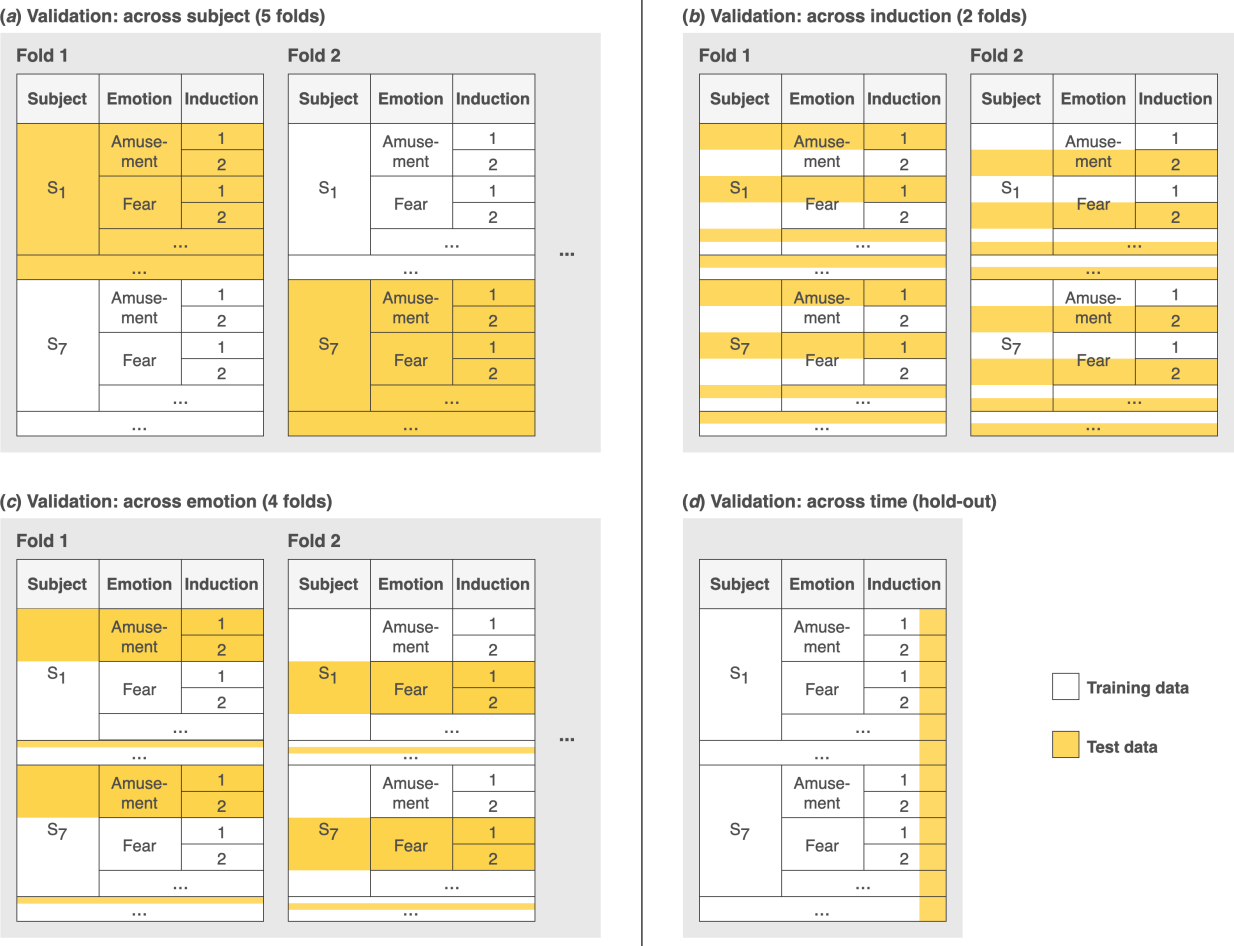
Overview of the four validation approaches. (*a-c*) Data were divided into subsets (folds). Models were iteratively trained on one set of folds (white) and tested on the remaining fold (yellow). (*d*) Models were trained on the beginning of all inductions and tested on later parts of the inductions.

To explore the implications of decisions about model training and testing, we examined four different approaches (figure 1):

(i) for *across-subject validation*, we used leave-*N*-subjects-out validation (figure 1*a*). Participants were randomly divided into five folds. Teams trained models on four folds and tested models on a fifth fold. This was repeated for each combination of folds;
(ii) for *across-emotion validation*, we used a leave-one-emotion-out validation approach (figure 1*c*). As a reminder, four emotions were targeted (via videos) in the original dataset: amusement, fear, boredom and relaxation. We created one fold for each targeted emotion (four folds total). Teams trained models on data from three targeted emotions and tested models on data from a fourth targeted emotion. This was repeated for each combination of folds;
(iii) for *across-induction validation*, we used a leave-one-video-out validation approach (figure 1*b*). As a reminder, each targeted emotion was induced through two different videos. For each targeted emotion, teams trained models on data from one video and tested models on data from the second video; and
(iv) for *across-time validation*, we used a hold-out validation approach focused on chronology (figure 1*d*). For each participant, we divided the data from each emotion induction into training and test sets based on time. Teams trained models on data from the beginning of the inductions and tested the models on data from the later parts of the inductions.

Notably, the four validation approaches represent what researchers might do to evaluate theoretical debates about the extent to which links between peripheral nervous system activity and affective experience are biologically innate (across-subject validation), emotion specific (across-emotion validation), similar across contexts (across-induction validation) and stable throughout the course of an emotional event (across-time validation) [30].

Test data files contained 30 s of affect reports (removed during testing) and their corresponding physiological recordings. Test data files also contained 10 s of physiological recordings that preceded and succeeded the affect report window. This allowed teams to potentially use short periods of past and future physiological data to predict affect reports. Teams were permitted to build different models for different validation approaches. Before submitting their models for evaluation on the final set of test data, teams were allowed to conduct up to three preliminary tests on a subset (50%) of test data. This approach is often used in machine learning to prevent overfitting on training data, thus promoting the generalizability of the models. Eight teams conducted these preliminary tests (see the electronic supplementary material, table S1).

The outcome of interest was the absolute value of the prediction error for self-reports of valence and arousal. We chose to focus on self-reports because it most closely maps onto a central construct of interest in emotion research: the *experience* of affective states. Prediction error was estimated separately for valence and arousal reports and then summed. To examine the impact of decisions about benchmarking, we compared teams to two simple baseline models: (i) a *random baseline model* that made random (within the range of the measure) predictions about self-reported valence and arousal reports; and (ii) a *mean baseline model* that uniformly predicted each rating based on the observed mean in the training dataset. However, other baseline models could certainly be considered, such as ones that calculate tailored subject-specific mean ratings, preserve first-order signal statistics and/or constrain continuity, temporal variability and autocorrelation.

For the three models judged to be the most promising, follow-up tests were conducted to evaluate the extent to which the models used peripheral nervous system features. For these tests, the peripheral nervous system data in the testing files were replaced with simulated physiological randomness: *N*(*μ* = 0, *σ* = 1).

## 3. Results

To examine each team’s accuracy, we used mixed-effect regression. Mixed-effect regression is a robust analytic approach that can be used to accommodate non-independent observations, which often lead to inflated error rates in traditional linear regression or data loss when averaged to avoid non-independence [37–39]. For each team, we regressed absolute prediction error as a function of: (i) whether the prediction came from a model developed by the team, the random baseline model or the mean baseline model; (ii) the validation approach used for testing; (iii) a higher-order interaction between model source and validation approach; and (iv) random intercepts for each subject and video in the dataset. Random intercepts were included to accommodate non-independent observations from the same participants and videos. For each validation approach, we used model-derived pairwise contrasts to estimate and test the significance of the mean difference (MD) in the absolute prediction error between the model developed by the team and each baseline model.

**Figure 2.**
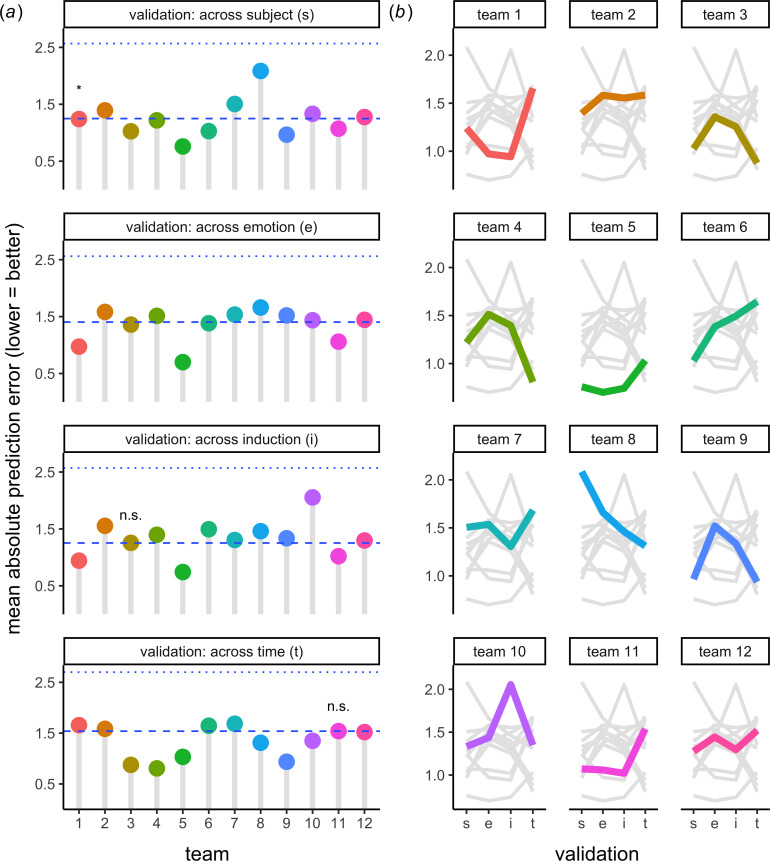
Differences in how machine learning models are developed, benchmarked and tested interactively shape conclusions about their ability to predict affect reports from physiology. (*a*) Absolute error of affect report predictions (*y*-axis) made by 12 teams’ models (*x*-axis). Validation approaches (panels) are visualized separately. Models are compared to a random baseline (upper dotted line) and mean baseline (lower dashed line). Note: *p* < 0.001 unless otherwise indicated; an asterisk denotes *p* < 0.05 for mean baseline comparison; *n.s.* denotes *p* > 0.05 for mean baseline comparison. (*b*) Re-illustration of teams’ (panel) prediction error (*y*-axis) across validation approaches (*x*-axis).

As illustrated in figure 2*a*, inferences about how well teams modelled affect reports depend on which baseline model is considered. When compared to a *random baseline model*, every team in every validation scenario had more accurate predictions (1.90 < MD > 0.48, all *z* > 130.76, all *p* < 0.001). However, teams did not always make more accurate predictions than a *mean baseline model*. Seven teams (58%) outperformed the mean baseline model in across-subject (0.50 < MD > 0.008, all *z* > 2.54, all *p* < 0.05) and across-time validation (0.74 < MD > 0.018, all *z* > 5.36, all *p* < 0.001); five teams (42%) outperformed the mean baseline model in across-emotion validation (0.71 < MD > 0.02, all *z* > 6.01, all *p* < 0.001); and three (25%) teams outperformed the mean baseline model in across-induction validation (0.52 < MD > 0.23, all *z* > 64.20, all *p* < 0.001).

As further illustrated in figure 2*b*, the manner in which prediction accuracy varied across validation approaches differed across teams. For example, team 1 achieved lower prediction error in across-subject versus across-time validation. Team 4 exhibited the opposite pattern. These results highlight how constraints on generalizability are *multiplicative*. Inferences depended on the interactive effect of decisions about (i) what models to deploy and (ii) how to evaluate those models.

### 3.1. Further evidence of the role of peripheral nervous system activity

The above results are consistent with previous claims that machine learning models can capture links between peripheral nervous system activity and affect reports [40,41]. However, although the models always outperformed random guessing, they did not always outperform a mean baseline model that uniformly predicted affect ratings as the mean of the ratings observed in the training dataset (figure 1*a*). This raises questions about the extent to which the models’ predictions were driven by the recovery of (i) theory-relevant links between peripheral nervous system activity and affect reports versus (ii) theory-irrelevant averages of affect reports in the training dataset.^1^

**Figure 3.**
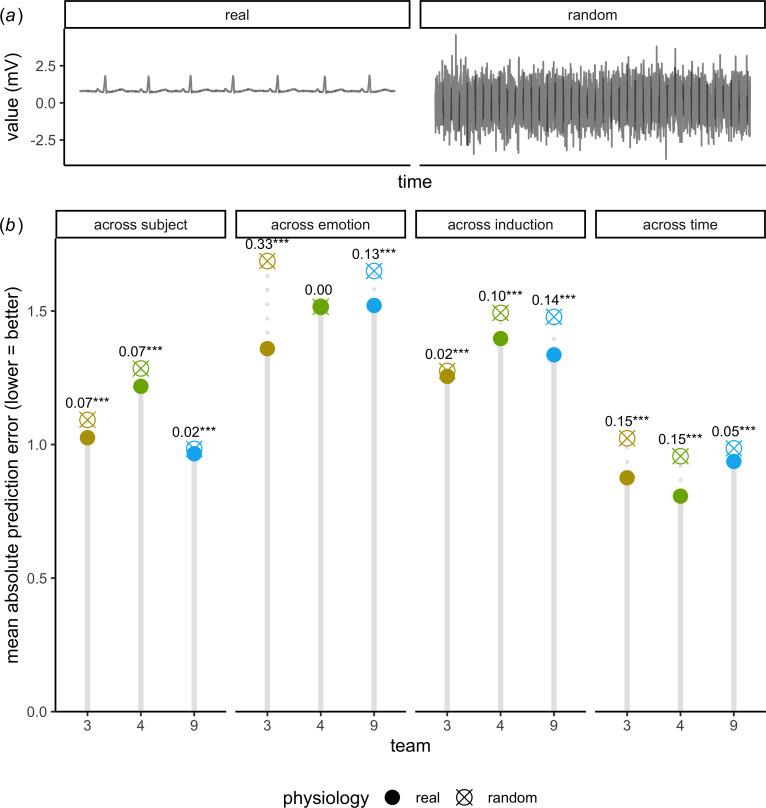
Three re-examined models typically exhibited less accuracy when re-tested on simulated physiological randomness. (*a*) Example of real electrocardiography signal versus simulated physiological randomness. (*b*) Absolute error of affect report predictions (*x*-axis) made by models developed by three teams (*y*-axis) when tested on real physiology (circle) versus simulated physiological randomness (crossed circle). Results are visualized separately for four validation approaches (panels). Significance levels correspond to mean differences in performance when a model was tested on real versus simulated physiological randomness. Note: ***p < 0.001.

One benefit of big team science is that it can be used to efficiently identify and probe particularly promising models. To further investigate the role of peripheral nervous system activity, competition organizers reviewed the openly available code for top-performing submissions and [subjectively] chose three that seemed particularly promising. The teams’ models were then re-tested on the same data with one change: measures of peripheral nervous system activity were replaced with simulated physiological randomness (figure 3*a*).

Using mixed-effect regression, we regressed each of the three team’s absolute prediction error as a function of (i) the validation approach used for testing, (ii) whether the test data contained real or simulated physiological randomness, (iii) a higher-order interaction between validation approach and whether the physiology input was real, and (iv) random intercepts for each subject and video in the dataset. In all but one case (MD = 0.00, *z* = −1.07, *p* = 0.29), the teams’ prediction accuracy decreased when tested on simulated physiological randomness (−0.33< MD > −0.018, all *z* < −6.96, all *p* < 0.001; figure 3). In other words, accuracy decreased in 93% of tests with simulated physiological randomness. These results provide evidence that most—but not all—high-performing models actually relied on the provided peripheral nervous system signals to predict affect reports. However, results also highlight the limits of what models learned from physiology, with real physiology only improving affect report predictions by a maximum of 0.33 points (on a 9-point scale).

## 4. Discussion

Taken together, the results of our big team effort reveal both the promises and limitations of machine learning efforts to model potentially complex physiological markers of affective experience. In four tests, models developed by 12 teams of researchers uniformly achieved higher accuracy than what would be expected by mere random guessing. About half the time, accuracy was also higher than what would be expected by merely using mean ratings from training datasets. Further evidence of the role of peripheral nervous system activity comes from follow-up tests with simulated physiological randomness, which nearly uniformly caused models from three selected teams to become less accurate. However, the magnitude of differences in prediction accuracy in many tests was small, illustrating extensive opportunity for future improvements.

More centrally, however, our results underscore challenges that past and future researchers face with commensurability and generalizability in research that seeks to use machine learning to predict and understand emotion [42,43]. Our results indicate that differences in how models are developed, benchmarked *and* tested can impact researchers’ conclusions. For instance, focusing on a random baseline (versus mean baseline) leads to a more optimistic interpretation of models’ accuracy—as does focusing on across-time validation (versus, e.g. across-emotion validation). These results have implications for ongoing discussions about the potential benefits (e.g. unobtrusive measurement of internal emotional states) and harms (e.g. inaccurate predictions) of emotion recognition technologies [44–47], such as those being pursued by a $20+ billion emotion AI industry [2].

Even more challenging is our observation that constraints on generalizability can be *multiplicative* (i.e. interactive) [43,48]. For example, the accuracy of predictions depended both on the modelling and validation approaches. However, it is inadvisable to make broad conclusions about the impact of any one of these decisions because they appeared to have an *interactive* effect on prediction accuracy. For example, team 1 achieved lower prediction error in across-subject versus across-time validation. Some researchers may be tempted to conclude that this provides evidence that links between peripheral nervous system activity and affect reports vary more within- versus between-persons. This conclusion, if true, would bolster claims that such links are biologically innate [49], but perhaps sensitive to context. This conclusion would also suggest that future research should focus less on the diversity of the sampled population and more on the diversity of the emotional contexts they encounter. However, such conclusions are premature when considering that differences in both modelling *and* validation approaches have *interactive* effects on prediction accuracy. Indeed, models developed by other teams (e.g. team 8) exhibited the opposite pattern: that prediction error is lower in across-time versus across-subject validation. Such results could be interpreted as evidence *against* claims of biological innateness and would underscore the importance of collecting diverse participant samples [50].

Multiplicative constraints on generalizability will be important to keep in mind as researchers increasingly use machine learning not only to predict emotion—but also to evaluate theoretical claims about its nature [31,51,52]. Our work focused on a specific theoretical issue in a specific methodological context: whether there are detectable links between physiological states and core affective experiences among participants in Germany in a stationary and controlled laboratory context. However, we suspect that our observed multiplicative constraints on generalizability will apply to other theoretical debates and methodological contexts in affective science. This includes debates about the number and discreteness of emotion categories [53,54], the extent to which their physiological correlates are invariant across different contexts and people [51], which physiological features (if any) are most robustly associated with specific emotional states [54,55], the utility of using wearable sensors to track emotional processes in non-laboratory contexts [20,56], and the performance of emotion recognition models when exposed to entirely new contexts (e.g. contexts with completely new data,^2^ such as real world contexts with ambulatory measures of physiology).

Although our work highlights challenges with commensurability and multiplicative constraints on generalizability, it also provides proof-of-concept for a potential methodological response: big team science [57–61]. Big team science effectively allowed us to use the wisdom-of-crowds to evaluate a fundamental theoretical question in affective science. Standardizing methodological decisions about data sources, benchmarks and testing procedures permitted cleaner comparisons of teams’ different approaches. Further introducing *systematic variation* in specific methodological decisions (e.g. testing procedures) allowed us to empirically examine the extent to which these decisions constrain the generalizability of inferences. Finally, requiring that teams make their materials and code openly available allowed us (and future researchers) to further inspect teams’ models, reproduce their solutions and identify approaches that seem most promising for follow-up research [62]. For example, although our analyses did not investigate the impact of specific methodological decisions, this remains a promising goal for future research.

Our examination of the use of big team science in machine learning research on emotion also yielded lessons about ways that future collaborative efforts can be improved and expanded. For instance, feasibility constraints prohibited the competition organizers from closely examining the code for all submissions, performing comprehensive evaluations of model behaviour and working closely with teams to navigate potential disagreements about the appropriateness of their methodology [63]. Expanding upon current guidelines for crowdsourced model development [64], developing protocols and best practices for *peer code review* could have better enabled the crowdsourcing of this task [65–68]. Such protocols, for example, could help researchers examine whether specific decisions predict variability in the results of machine learning efforts [69]. Teams that entered the competition also faced resource constraints, with many expressing that they would have benefited from (i) more time to work on the challenge, (ii) datasets with more peripheral nervous system measures, observations and cultural variability, and/or (iii) access to more powerful computing resources. Recent pushes to collaborate on dataset development [57–60] and provide access to shared computing resources [70] may prove instrumental in helping researchers overcome those barriers.

Despite existing constraints, our work raises an exciting possibility: advancements in machine learning and collaborative research methods provide researchers with new tools for tackling ultra-complex questions in affective science and beyond. However, when doing so, researchers will have to grapple with multiplicative constraints on generalizability.

## Data Availability

Data, materials and code are openly available at [71]. Supplementary material is available online [72].

## Footnotes

^1^
One challenge with connecting machine learning to theoretical debates about emotion physiology is model interpretability; i.e. it is unclear if successful predictions are driven by (i) theory-relevant links between peripheral nervous system and affect reports, and (ii) theory-irrelevant averages of affect reports in the training dataset. To illustrate this point, consider a classic linear regression context. Even without modelling physiology-related parameters, an intercept-only regression can often make reasonable predictions about participants’ affect ratings (if mean affect ratings captured by the model intercept are similar in training and test data). To more specifically make claims about physiology, linear links between physiology parameters and affect ratings would be formally modelled and inspected. Unlike linear regression, many machine learning methods (e.g. deep learning) do not fit interpretable parameters. Thus, researchers often need to deploy supplemental investigations to substantiate claims about what their models learned (if anything) about emotion physiology.

^2^
When the competition was designed, the organizers searched for an external dataset with similar methodological characteristics (moment-to-moment measures of core affect and a similar suite of physiological measures). Such efforts were not successful (even after reaching out to the developers of the dataset that was used).
